# Data linkage in medical science using the resource description framework: the AVERT model

**DOI:** 10.12688/hrbopenres.12851.2

**Published:** 2019-03-14

**Authors:** Brian P Reddy, Brett Houlding, Lucy Hederman, Mark Canney, Christophe Debruyne, Ciaran O'Brien, Alan Meehan, Declan O'Sullivan, Mark A Little

**Affiliations:** 1 Trinity Health Kidney Centre, Tallaght Hospital, Dublin, Ireland; 2 ADAPT Centre for Digital Content, University of Dublin, Dublin, Ireland; 3 Health Economics Policy and Evaluation Centre, National University of Ireland, Galway, Galway, Ireland; 4 School of Computer Science and Statistics, University of Dublin, Dublin, Ireland; 5 Vrije Universiteit Brussel, Brussles, Belgium; 6 Irish Centre for Vascular Biology, University of Dublin, Dublin, Ireland

**Keywords:** evidence-based medicine; information and knowledge management; data security and confidentiality; resource description framework; semantic web; linked data; electronic health records

## Abstract

There is an ongoing challenge as to how best manage and understand ‘big data’ in precision medicine settings. This paper describes the potential for a Linked Data approach, using a Resource Description Framework (RDF) model, to combine multiple datasets with temporal and spatial elements of varying dimensionality. This “AVERT model” provides a framework for converting multiple standalone files of various formats, from both clinical and environmental settings, into a single data source. This data source can thereafter be queried effectively, shared with outside parties, more easily understood by multiple stakeholders using standardized vocabularies, incorporating provenance metadata and supporting temporo-spatial reasoning. The approach has further advantages in terms of data sharing, security and subsequent analysis. We use a case study relating to anti-Glomerular Basement Membrane (GBM) disease, a rare autoimmune condition, to illustrate a technical proof of concept for the AVERT model.

## Introduction

The availability of data has been growing exponentially in recent years ^1^. This poses practical challenges with regard to seemingly prosaic problems such as how to store the data, as well as more fundamental issues such as how best to organise datasets to facilitate subsequent analyses. In health settings, there are further specific challenges in management of sensitive patient data in the context of the introduction of the European Union General Data Protection Regulation (GDPR) ^2^.

Anti-glomerular basement membrane (anti-GBM) disease is a rare autoimmune disease that is characterised by rapidly progressive kidney failure and bleeding from the lungs. It is caused by the development of an abnormal immune response to a protein that is expressed in these organs ^3^. It affects about 1 person per million per year and has a poor prognosis if not treated early. We have previously identified geographic and temporal clusters, strongly suggesting an environmental trigger ^4^. However, the specific causes of these clusters have not been investigated.

Autoimmune diseases generally occur when an individual with a genetic predisposition encounters something in their environment that triggers the immune system. Japanese clusters of diagnoses of Kawasaki disease, a related autoimmune disease, have been shown to exhibit clear links with the tropospheric wind direction which carries a specific species of *Candida* fungus from China ^5, 6^. It is therefore plausible that occurrence of anti-GBM disease could similarly relate to weather, pollution and/or infectious disease conditions. The rarity of this condition precludes use of classical case-control studies, mandating the development of novel approaches.

Attempting to identify potential environmental triggers of anti-GBM disease created the challenge of organising the datasets in a systematic and open manner, and of merging multiple environmental and patient-level datasets. We describe here the informatics techniques adopted to address this, developed as part of a larger project: Autoimmune Relapse Prediction using Multiple Parallel Data Sources, given the acronym “AVERT”. We used a series of steps to transform heterogenous data (most with a temporo-spatial component) from a variety of different formats into a single queryable data source. This single data source facilitates further insights through data enrichment, eases the application of machine learning approaches, allows for accurate data provenance and supports scientific data management best practice according to the FAIR open data source principles ^7^. The Resource Description Framework (RDF) data model ^8^ proved an ideal framework for managing the data integration process. The aim of this paper is to provide a technical proof of concept of the model used, using the example of anti-GBM disease, which has potential applicability in many health informatics settings. The next section sets out the context for this work and introduces concepts which may be familiar only to computer scientists.

## Background

Evidence-based approaches to medical decision making rely on robust data and evidence ^9– 11^. The quantity of potentially usable data that may inform healthcare questions is increasing rapidly. However, significant practical challenges in accessing these data remain, which are frequently unstructured, and in assembling what is available into “sufficiently expressive and flexible representations” ^12^ in order to facilitate further analysis.

The *Semantic Web* is an initiative to represent ‘resources’ (i.e. documents and things represented by these documents) on the World Wide Web in such a way as to facilitate data linkage and processing, thereby “better enabling computers and people to work in cooperation” ^13^. This allows computer-based agents to ‘understand’ data using ontologies ^14^, which provide a vocabulary of basic concepts related to each other within a specific area of interest ^15^ and describe concepts in codified, easily understood definitions. These vocabularies allow for lateral homonyms (i.e. as with a thesaurus) and the creation of hierarchical relationships ^16^.

*Linked Data* can be considered as the combined set of best practice techniques to capitalise on the Semantic Web. Berners-Lee proposed four principles in order to achieve this:

1. Use Uniform Resource Identifiers (URIs) as names for things.
2. Use Hypertext Transfer Protocol (HTTP) URIs so that people can look up those names.
3. When someone looks up a URI, provide useful information, using the standards – for example, RDF and SPARQL (SPARQL Protocol and RDF Query Language).
4. Include links to other URIs, so that they can discover more things.

A URI is a string of ASCII characters that can identify a unique resource, which could be a digital representation such as a song or a document, or a representation of a tangible physical object such as a person or a place. HTTP protocols allow for the URIs to be dereferenceable, meaning users can follow the URI link of a resource and retrieve information on that resource ^17^.

The *Resource Description Framework (RDF)*, is a graph based data model that allows data to be represented in the form of a triple – comprising a subject, predicate and object (for example, “Patient 1”-“has date of birth”-“20-10-1985”). When used in conjunction with ontology building languages, such as RDFS and OWL (see below) it is possible to build rich, structured, semantic models to describe data:

1. *RDF Schema (RDFS*) ^18^ is a collection of terms (classes and properties) that can be used to build simple ontologies for describing domains of knowledge. It allows basic axioms to be declared about data which supports limited reasoning over the data.
2. The *Web Ontology Language (OWL)* ^19^ is another collection of terms for building ontologies; however, it is more expressive than RDFS and allows declaration of more complex axioms. These complex axioms facilitate more in depth reasoning and inconsistency checking over data.

The RDF model, RDFS and OWL are all W3C standards. These standards are set by the World Wide Web Consortium, an organisation which develops protocols and guidelines to “ ensure the long-term growth of the Web”. As a W3C recommendation, RDF comes with other specific advantages in terms of recognition and compatibility, including packages in the R statistical software environment, such as Redland ^20^, to allow interaction with the data. In the example above a previously described and well-known ontology definition of “has date of birth” (e.g. schema:birthDate) could be used, making the triple easily understandable.

A database that stores RDF data is known as a *triplestore*. Triplestores facilitate efficient data storage of multiple sets of RDF data, which would otherwise prove cumbersome. Most triplestores provide a means to access data through querying. Querying is done with the SPARQL query language, the W3C recommended query language for RDF data.

*GeoSPARQL* (an Open Geospatial Consortium standard) allows for “common representation of geospatial RDF data and the ability to query and filter on the relationships between geospatial entities”. It provides an ontology for representing geospatial RDF data, but also an extension of the SPARQL query language to formulate geospatial queries (e.g., to retrieve all cities in a particular country, or to identify all patients within a given radius). Therefore, the GeoSPARQL standard allows for more powerful querying of spatial data.

By recording the data’s provenance and metadata, relationships between fields can be explicitly highlighted and understood more easily, showing how rules were derived, by whom and when. Such provenance is vital given the necessarily limited human oversight when using machine learning techniques, and to ensure traceability between the producers and consumers of the derived information ^21^. The PROV Ontology (PROV-O) ^22^ is another W3C standard which has been designed to represent provenance information in this way. This is of increasing importance in the context of Europe’s upcoming General Data Protection Regulation (GDPR) ^23^.

Tabular data (e.g. CSV and TSV files) can be transformed into RDF format through a process known as “uplift” ^14^. This process specifies explicitly how data within a table should be represented in RDF, and how it should be described according to an ontology. Uplift is carried out using R2RML (another W3C recommendation ^22^), which is a language for expressing customized mappings from tabular form and relational databases into RDF. Such RDF files can be enriched through the linking of datasets. For example, using GeoSPARQL, one can ascertain which county a given set of coordinates is within, and then link to that county with the coordinate triple in the RDF file. If required, this enriched dataset can be converted back into tabular format (e.g. CSV), which would now include this county location data. Transformation of RDF data back into tabular format is called “downlift” ^14^, and in many cases this step is required to allow for further data analysis by many statistical software applications.

## Development and methods

While clinical and environmental datasets could in principle be linked in a single flat file or relational database using temporo-spatial fields, given their large and disparate nature, a systematic approach based on RDF to manage their integration is more effective. This allows temporal or spatial data of differing granularities to be stored in their original format, helping to document their provenance. For example, three different datasets may be available weekly, daily and hourly – in RDF they can be stored in their original format, whereas in a single tabular file human judgement would be required as to how to ‘fill in the gaps’. RDF approaches also facilitate sharing of the data to support similar geo-medical research in the future. Models of meteorological and pollution conditions ( Table 1) were identified and included in subsequent analyses, alongside two live national datasets on notifiable disease infection (the Computerised Infectious Disease Reporting [CIDR] and Influenza-like illnesses [ILI] databases).

**Table 1.** Initial datasets uplifted into RDF triple store. *Computerised Infectious Disease Reporting, ~Local Health Organisation, #Influenza-like illness, +European Centre for Medium-Range Weather Forecasts, = European Monitoring and Evaluation Programme, > Meteorological Synthesizing Centre - West. NA = Not applicable

Dataset	Temporal data level	Geospatial data level	Initial Size	Format	Source	Freely available online?
Clinical patient description	Daily	Town/Townland	14KB	CSV	Medical records	No
CIDR *	Weekly	LHO ~ area	286KB	CSV	Health Service Executive	No – required formal agreement
ILI ^#^	Weekly	National	15KB	CSV	Health Service Executive	No – required formal agreement
Weather1	Daily	Linked to weather station location file	25MB (cumulative)	One CSV file per station	Met Éireann	Yes
Weather station location	NA	Coordinates	3KB	CSV	Met Éireann	Yes
Weather2	Daily	0.75° *0.75° grid	4.72GB	netCDF	Sample from ECWMF ^+^ ERA - Interim dataset	Yes
Pollution	Daily	50 *50km grid	8.75GB (cumulative)	One netCDF file per year	EMEP ^=^ MSC-W ^>^	Yes
Ordnance Survey of Ireland	NA	Authoritative boundaries at various levels: Barony; City/county council; County; Electoral division; Local electoral area; Municipal district; Parish; Rural area; Townland	419 MB	RDF	data.geohive.ie	Yes

### Step by step approach to model building

**Figure 1.**
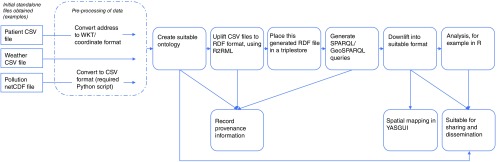
The approach to transform siloed tabular datasets into RDF, and back into enriched file for analyses, Adapted from Debruyne *et al.* ^6^. Only a sample of files used are shown.

Figure 1 illustrates the series of steps in development of the AVERT model, which were adopted to: obtain the relevant datasets, represent them in RDF, enrich the data using different processes, and then represent the enriched data in a format that would enable analysis.

***Step 1: Obtaining and understanding datasets.*** Gaining ready and regular access to relevant datasets is a recurring, and underappreciated, challenge in analytics projects. It requires background knowledge and understanding of which datasets are available, permission for their use where required, careful selection of appropriate data sources, and the ability to handle data of differing formats. The datasets transformed into RDF in this case study are summarised in Table 1. Patient-level data was defined as described previously ^4^. Data that describe elements of a person’s environment, on the other hand, were based upon external datasets, including:

- data directly recorded from weather stations (Weather1);
- modelled estimates of weather and pollution (Weather2, Pollution);
- counts (CIDR) and rates (ILI) of infectious diseases in specific areas.

Most datasets had some form of temporal component, albeit at different granularities, and all had some form of location encoded. These different geospatial data levels are more challenging to reconcile than temporal ones given the wide range of formats and concepts used.

Weather stations have a location (latitude and longitute) collated from the Irish weather service (Met Éireann). Historical daily weather datasets were available for download for each weather station, with variables such as precipitation levels, mean wind speed and max/min temperature included.

Both European Centre for Medium-Range Weather Forecasts (ECWMF) and European Monitoring and Evaluation Programme (EMEP) datasets were downloaded in NetCDF (Network Common Data Format) format, initially at a European continent-wide level. Such datasets are a set of interfaces for array-oriented data access and for storing and retrieving multidimensional data, which are common in meteorological, climate and GIS studies; they are typically very large and require specialist software to open. These NetCDF files subsequently needed to be transformed into CSV format before uplifting; this transformation was carried out using a Python script which made use of a specific library for accessing NetCDF encoded data. As our study was only concerned with Ireland, only relevant coordinates were transformed into CSV. As a result, their filesizes reduced considerably, from 8.7GB and 4.7GB to 76MB and 23MB respectively.

While these datasets were publicly available, others required liaison with public health officials in order to gain access to them. Infectious disease data (CIDR) and Influenza Like Illness (ILI) location data are not encoded in any standard geospatial format. CIDR data ^24^ are reported weekly at both “Local Health Office” (LHO) level – which broadly corresponds to county level (though counties Dublin and Cork were divided further). The ILI dataset is compiled from a sample of family doctors around the country to provide an estimate of the national near-real time weekly rate of presentation of respiratory syndromes that could be influenza, and cannot be drilled down to at a more local level.

Authoritative linked data borders of several Irish geographic level geospatial units have been published online by the Ordnance Survey of Ireland (OSI), such as those of counties, electoral districts (small sub-divisions of counties) and so on. These boundary data (available here) was used to help with the grouping of data on a spatial level (e.g. CIDR data is reported at the county level, weather and pollution data only have latitude and longitude coordinates). The OSI data allows, for example, the identification of all weather and pollution data for a patient’s county.

Because of the presence of sensitive data, the patient dataset had been de-identified, and patient addresses were only available to analysts at town/townland (a smaller village-scale) level. This location was approximated to a single point (latitude and longitude coordinate), using the centroid of the townland as found in Google Maps. LHO data were not suitable to represent as a single point, and not all their borders were available in the OSI boundary dataset. ILI data, on the other hand, was only available at a national level. While this meant that no manual construction of areas was required, it meant that more granular spatial analyses were not possible.

**Figure 2.**
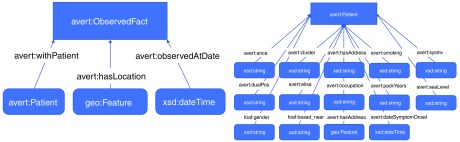
Model of links between diagnosis (“observed fact”) and other fields in patient dataset, and ontologies used to map these. Prefix definitions - avert: < http://data.avert.ie/avert#>; geo: < http://www.opengis.net/ont/geosparql#>; xsd: < http://www.w3.org/2001/XMLSchema#>.

***Step 2: Knowledge representation.*** Where large amounts of data are available and necessary, it becomes crucial to consider how best to organise the data into a suitable format to support subsequent reliable and scalable statistical analyses ( Figure 2). Taking time to ensure that the analyst has fully understood and explicitly described the data landscape has obvious similarities to soft-systems methodologies in operational research ^25^.

Entity-relationship diagrams are a useful way of structuring the underlying relationships between fields, and can help to clarify the most appropriate ontologies to use to allow meaningful data linkage. Existing ontologies can, to a certain extent, be mixed and matched to create a set of definitions that fit the data’s needs. There are advantages and disadvantages of using creating bespoke ontologies (assuming the choice is available), summarised in Table 2.

**Table 2.** Advantages and disadvantages of using an existing ontology or creating a bespoke one.

	Use existing Ontology	Create Bespoke Ontology
**Advantages**	Reusing existing (possibly well known) ontologies will make our data more interoperable which help with the “Interoperable” clause of the FAIR principles.	Creation of a bespoke ontology allows us to model our data in an efficient manner.
**Disadvantages**	Existing ontologies may not offer the most efficient way to model our data – increasing complexity which leads to reduced performance in data retrieval.	Creation of a bespoke ontology would reduce interoperability of our data.

We attempted to use an ‘in between’ approach that utilised existing ontologies where possible, and referred to existing ontologies to improve the data’s interoperability. The derived ontology required using multiple levels. Each anti-GBM diagnosis (our ontology deemed this an ‘observed fact’) is associated with a date, a location and other data specific to the individual patient. For patients themselves, a well-known generic ontology for describing people – FOAF (“ Friend of a friend” ) – was used to specify certain attributes, such as gender. However, others, such as smoking status, occupation category or results of medical tests, are not covered by this and hence were specified in an ontology designed specifically for this study.

***Step 3: Uplift.*** An R2RML declarative mapping was used to tranform each CSV file into RDF format. This explicitly maps the meaning of data fields, following the ontologic model developed in the prior stage. Data can also be formatted at this stage to align with existing standards; for example, in the anti-GBM study dates were converted to standard yyyy-MM-dd format at this stage, and field definitions were clarified, such as Gender=0 in the patient CSV file being defined as ‘Female’.

In the ontology depicted in Figure 2, ‘observed fact’ comprises dateTime, Location, and Patient. Each of these fields is themselves defined modularly and in reference to each other, with location for example being defined as being made up of the longitude and latitude fields of the patient dataset.

From there, each predicate must be defined. For example, gender is defined as *foaf:gender*. Because FOAF is a well known ontology, there should be no ambiguity subsequently as to what definition of ‘female’, for example, is used if the data is shared in future. This process was carried out for each field that was intended to be transformed to RDF. Once uplifted to RDF, the data consists of a series of triples. For example, a weather station (with the URI “http://data.avert.ie/weather_station/Mullingar%20Automatic%20Weather%20Station%(AWS)">http://data.avert.ie/weather_station/Mullingar%20Automatic%20Weather%20Station%(AWS)”) is both a ‘Feature’ (with the geometry (i.e. WKT location) of -7.362222222, 53.53722222) and a ‘Weather Station’ (with the label “Mullingar Automatic Weather Station (AWS)”). Each of these pieces of information constitutes a queryable triple related to the station, and which can in turn be related to other datasets. The number of triples thus grows rapidly, as does their analytical power through such linking.

***Step 4: Enriching the RDF data.*** When in RDF format, the data can thereafter be further processed in order for it to be enriched by creating ontological relationships that add depth and meaning to the data. For example, the closest weather station to each patient could be identified using a GeoSPARQL query containing a geospatial function (which is processing intensive). The results of such a query can then be inputted to the data so there is now a direct link between patients and weather stations – reducing the need to perform another geospatial function in order to determine this information.

**Figure 3.**
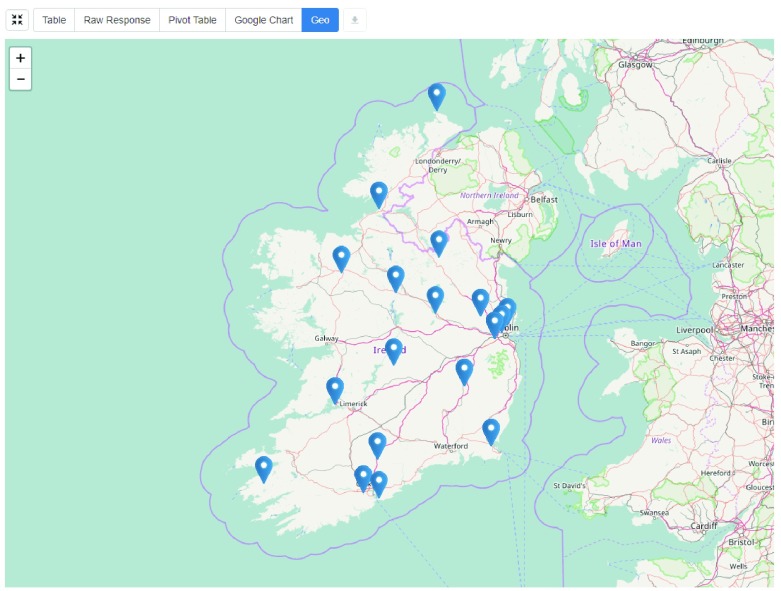
Locations of weather stations used for analysis, generated using GeoSPARQL analysis of RDF triplestore, and visualised using YasGUI.

Data for associated weather stations can thereafter be more easily accessed for each patient, to allow analysis of the weather conditions for each person’s address in the period prior to diagnosis. The locations of weather stations included in the analysis are shown in Figure 3, visualised on the *YasGUI* web client ^26^, which allows geographic data to be visually represented on a map.

Since we were using the OSI boundary dataset, and since most of the other datasets used contained a geospatial element (usually a point), we used GeoSPARQL for subsequent querying at various levels, for example:

- Geographical; e.g. “Given a patient’s location, find the region (county, townland, etc.) in which that patient resides”;
- Temporo-spatial; e.g. “Retrieve weather and pollution records within the specific region of the country over a specific date range.”

Complex federated queries; e.g. “Given each patient’s location, retrieve the nearest weather and pollution readings within a specific date range around the patient’s diagnosis date, but excluding patients with a specific comorbidity”. YasGUI visualisations of this data are possible for such queries, potentially generating new insights. The OSI border dataset allow for queries to be run on the data which would otherwise not be practicable, and across multiple datasets. The previous study of these anti-GBM cases ^4^ carried out the analysis at the level of counties, but the AVERT model allows for the investigation of whether clusters occurred in smaller areas, or straddled county boundaries, for example. The time, date and identity of the author of the query can be recorded using the PROV-O ontology, as can similar information regarding the mapping and links to underlying models.

***Step 5: Downlift and analyses.*** Once all data has been transformed into RDF and enriched, it can be explored in its entirety. This exploration may lead to specific data that investigators wish to perform a detailed analysis over. In some situations, RDF may not be a suitable form to perform this analysis, therefore it must be downlifted to a less expressive form such as CSV. In the case study, an enriched CSV file was created from the RDF data, which could subsequently be easily analysed in R. For each patient record, prior weather and pollution data could be collated into a single file. In general, after one round of analyses, modellers may subsequently wish to alter which fields to analyse, the fields’ definitions, revisit queries, or may realise that new interpretations of how the data were mapped are necessary. Thereafter, the analysis may become an iterative process until a final statistical model is agreed upon. Alternative approaches to CSV files - such as Jupyter notebooks - may facilitate better retention of provenance data, and may become more common in future.

## Discussion

This paper has demonstrated a pragmatic standards-based solution to integrating temporo-spatial environmental data with patient-level information in order to address an epidemiological research question. The technique is modular, allowing additional data sources, such as smartphone derived telemetry, biomarker information or other environmental factors, such as radon exposure, to be incorporated later, and can be applied to a diverse range of applications.

Several prior publications have addressed the use of RDF approaches to improve biomedical data annotation. Mayer *et al*., for example, use an RDF schema to assist in labelling the quality standards of medical websites ^27^. Another paper by Mayer *et al*. describes a platform to automatically generate metadata descriptions that can be used to label the trustworthiness of the content of medical websites ^28^. This metadata can be accessed through standard search engines, and the fact that the data are machine readable allows for more targeted querying, as well as potentially advancing interoperability.

The Open European Nephrology Science Centre project (OpEN.SC) study ^29^ takes this further, using an RDF approach to generate a common data model from multiple standalone clinical datasets, and to facilitate querying across these by researchers. Datasets were derived from patients undergoing kidney transplantation across 18 sites, each with their own data formats and structures. These were subsequently uplifted into RDF. The authors’ aim was to have a common data model for clinical data, then to integrate the data and provide a convenient intelligent retrieval interface. This has much in common with the Bio2RDF project ^30^, which attempted to integrate multiple biological data sources using semantic web technologies. They built a large triplestore describing human and mouse genomes, and provide a case study of how to perform a federated query across these to identify diseases associated with individual genes on a specific pathway. A further paper by Hochheiser *et al*. ^12^ describes the process of mapping clinical datasets into a computational infrastructure, allowing for future extraction and examination of patient-level data at various levels of abstraction. One of the key advances of the AVERT model compared to these papers is that it is not confined to clinical settings, and that linking these with environmental datasets requires more explicit consideration of time and place, and hence temporo-spatial reasoning.

Other studies have addressed the related issues of interoperability and data sharing over recent years, and argued firmly for them to be considered explicitly. The FAIR (findability, accessibility, interoperability and reuse) data principles ^7^ provide a framework for sharing data in a way that maximises its use and reuse. They emphasise the importance of allowing machines to automatically discover, process and integrate digital objects. Suitable approaches to data management include, but are not confined to, RDF; the guidelines are not proscriptive in this regard. Instead, they advocate that data siloes can be searched and integrated, building towards a future where machines may begin to “understand” and “make a useful decision regarding data it has not encountered before”. Sansone *et al*., in a paper about the ISA (investigation/study/assay) metadata framework ^31^ also argue for the inseparability of data management and data sharing, and the benefits that could be derived from a “data communing” culture. As with the FAIR principles and the OpEN.SC study, the ISA paper emphasises the risk that smaller projects may become data siloes if specific efforts are not made to address interoperability. Data provenance is also of utmost importance as the environment moves towards a future of “machine actionability” ^7^. In this regard, the OpEN.SC study highlighted that RDF has specific provenance strengths as it “is particularly useful for storing metadata about shared resources” ^29^.

One innovative approach that matched high resolution geo-location data and real-time health data was the Flutrack study ^32^, which mapped self-diagnoses of influenza-like illness on Twitter. The authors had found that open-source systems and shared methodologies were not widely used in health informatics and public health, as they are at “an early stage in the development of modular and interoperable practices”. The data protection issues surrounding handling of patient data also present a very substantial obstacle to progress in this direction. They are nonetheless hopeful that such trends will continue to develop in future, as there is no reason (or moral justification) to try to maximise customer lock-in in public health settings. They advocate for increased use of such technologies to allow the development of “an ecosystem of applications and services”.

**Figure 4.**
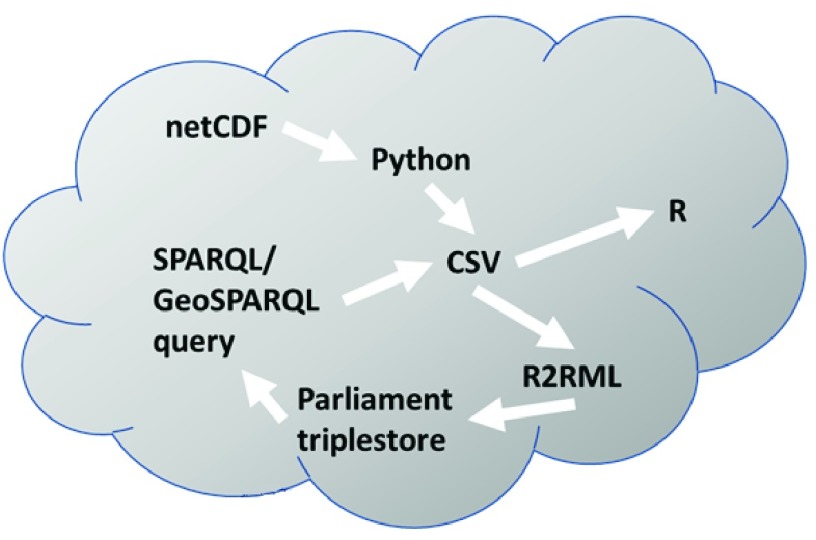
AVERT ecosystem and its “life course”.

Our proposed AVERT model provides a framework for highlighting how the existing “ecosystem” of languages, software and W3C standards can be combined into a package of approaches, and to describe the advantages of doing so, shown in Figure 4. This may be considered a step towards the aims of approach of the Hochheiser project ^12^, which attempted “to develop a generalizable computational infrastructure that will facilitate the extraction, manipulation, and use of these deep phenotypes, combining them with genomic data to drive discovery and precision medicine”. This ‘package of packages’ can be used to integrate standalone files, query across them and generate new analysable, enriched files featuring the most relevant variables in a common format. Furthermore, the AVERT model attempts to do so while adhering to the FAIR principles. The model was developed as part of a specific study, described in the section below, but will have applicability in broader health informatics settings. The model developed organically, with packages chosen based upon what we believed would work for the specific circumstances of the case study. As such it was not intended to be a systematic process, and did not investigate or list all potential such approaches. For other studies that intend to achieve similar outcomes in different circumstances, pragmatism and human judgement may be similarly required to ensure that the most appropriate packages are used for that data environment.

A key challenge was understanding how best to facilitate temporal and spatial reasoning, i.e. representing the target data sources in four dimensions. But tensions also exist between ensuring security of confidential patient data, whilst being committed to the principles of open data, data sharing, re-use of data resources and research transparency. While the open linked data principle can be considered a public good, the fact that it allows data to be more easily accessed and understood may create unintended consequences. Previously, sensitive data may have been unwittingly protected due to the difficulty of accessing it and linking across data siloes. As technology breaks these walls down, data managers will need to seriously consider what issues can be traded off and where suitable firewalls need to be created. A clear data management plan is strongly advisable in such circumstances to minimise the risk of accidental sharing of private information. In the longer term, common standards (possibly including legislation) for the sharing of health data should continue to be developed in order to facilitate a more predictable and secure environment to do so.

With regards to the case study, despite de-identification of the patient data, potentially distinguishing features remain, such as the patient’s date of birth or location. Given the rarity of anti-GBM disease it would be straightforward to re-identify specific patients given this information. Even if these fields are removed, linked data such as nearest weather station may give enough background information for data to be compromised in this way. Furthermore, it is difficult to envisage a flawless approach for linking data. For example, the approach described in ‘Step 4’ of linking patient environmental conditions with those of the nearest weather stations using GeoSPARQL and OSI geospatial data was potentially limited, although there is some value in such parsimony and in using only the ‘gold-standard’ of direct measurements taken at such locations. As the mapping algorithm was written in-house, the limitations and provenance of the model could at least be fully understood, and revised later if necessary. In contrast, the alternative approach of using the imputed estimates of weather available from the ECWMF would mean that these must be taken at face value (given that they were developed externally). This is counter to the principle of data provenance. On the other hand, these may well be more reliable than the ‘nearest weather station’ approach, are available at much finer granularity and have been validated. There is therefore an inevitable tension between deciding which dataset is more trustworthy.

Commonly agreed interoperable standards could be used, to develop a longer term “information commons” approach to facilitate further understanding of anti-GBM disease (or other diseases) by other researchers ^31^. Provenance will play a role here, helping, not only to engender trust in highlighting the links between abstracted models and source data ^12^, but also to describe how analyses were carried out and reducing the ‘black box’ risks when using machine learning techniques. However, this will not necessarily answer the question of what constitutes a more ‘trustworthy’ source in every setting.

In contrast with the prior literature, this project had the additional challenge of incorporating environmental conditions alongside clinical data, and using these data in predictive models. Where possible, all representations of data have followed existing W3C and community standards, in order to ensure data compatibility, understanding and face validity. Allowing sharing of these data may help to derive solutions to such issues more quickly through collaboration with external groups, or even independently. RDF approaches also facilitate more meaningful querying than would otherwise be possible ^28, 33^, and subsequently more meaningful statistical and machine learning analyses.

## Conclusions

We have described the development of a model which can be used to uplift tabular data (from a variety of sources) into a common RDF format. From this it can:

1. Be converted back into a tabular format via downlifting, enriched by incorporation of external data sources and reasoning algorithms.
2. Be managed in a codified format that follows well understood ontologies, facilitating sharing and understanding by both external groups and machine learning scenarios.

A clear advantage of the AVERT model when compared to standalone, siloed tabular files is that the integration of data in RDF, alongside the use of SPARQL allows complex querying of data to be much more easy to understand and manage. While some matching of tabular files in various granularities may be possible across CSV files, federated queries would eventually become impractical as they became more complex. Merging datasets in the manner espoused in this paper should instead help to ensure that the data is managed effectively the risk of human error is reduced. Once data are linked, it may lead to new opportunities for understanding causal mechanisms. Some of these may be simple tools, such as facilitation of visualisations, or more complex, such as supporting the use of machine learning approaches.

## Software availability

All software tools are listed in Table 3 below.

**Table 3.** All software tool used.

Tool	Link	License
Parliament Triplestore	http://semwebcentral.org/frs/?group_id=159	BSD License
R2RML Implementation	https://opengogs.adaptcentre.ie/debruync/r2rml	MIT License
Python conversion Scripts	https://www.scss.tcd.ie/~almeehan/avert/python_scripts/	GNU General Public License

Archived version of the Python conversion scripts are available from Zenodo: http://doi.org/10.5281/zenodo.1345525 ^34^

Scripts available under a CC BY-SA 4.0 licence

## Data availability

A description of all datasets used, including their availability and how they can be accessed, is presented in Table 4.

**Table 4.** All datasets with availability and access information.

Dataset	Organisation	Description	Availability	To access
Clinical patient description	Rare Kidney Disease Registry & Biobank	Patient-specific characteristics for all cases of anti-GBM in Ireland over the study period	While the underlying patient data is de-identified, because of the rareness of the condition, it is not possible in practice to fully anonymise the dataset. Individuals could potentially be re-identified quite easily, through variables such their diagnosis date or location (which, even if removed could be surmised from links with weather stations).	Requests to share aggregated information will be considered on a case by case basis. Contact Principal Investigator: mlittle@tcd.ie
CIDR	Health Protection Surveillance Centre, Health Service Executive	Shared national information system to manage surveillance and control of infectious diseases	Data requests are assessed on a case-by-case basis.	Contact hpsc@hse.ie
ILI	Health Protection Surveillance Centre, Health Service Executive	Irish sentinel GP influenza- like illness consultation rates per 100,000 population by week	Data are published in weekly reports.	http://www.hpsc.ie/a-z/respiratory/influenza/ seasonalinfluenza/surveillance/influenzasurveillancereports/
Weather1	Met Éireann	Historical datasets	Free to download	https://www.met.ie/climate/available-data/historical-data
Weather station location	Chronic disease informatics group, TCD	File manually created by this paper’s authors using latitude and longitudes given for each weather station in Met Éireann historical datasets	Free to download	https://www.scss.tcd.ie/~almeehan/avert/Weather_ Observing_Stations.xlsx
Weather2	European Centre for Medium-Range Weather Forecasts (ECMWF)	ERA-Interim dataset	Free to download	http://apps.ecmwf.int/datasets/data/interim-full-daily/ levtype=sfc/
Pollution	European Monitoring and Evaluation Programme (EMEP)	MSC-W	Free to download	http://emep.int/mscw/index_mscw.html
Ordnance Survey of Ireland	Ordnance survey of Ireland	Linked Data Fragments client	Free to query	http://client.geohive.ie/
