# Supplementary material for: Data linkage in medical science using the resource description framework: the AVERT model
Source: HRB Open Res. 2019 Mar 14;1:20. Originally published 2018 Aug 29. [Version 2] doi: 10.12688/hrbopenres.12851.2 (PMC6973528; doi:10.12688/hrbopenres.12851.2)
Supplement: Peer review history

# Peer review: Reviewer response for version 2

**DOI:** 10.21956/hrbopenres.13974.r26541

**Published:** 2019-04-10

**Authors:** Helena F. Deus

**Affiliations:** 1 Elsevier, Cambridge, MA, USA

The paper is acceptable for indexing. However, the reference link was broken when I tried to access it: http://data.avert.ie/.

I confirm that I have read this submission and believe that I have an appropriate level of expertise to confirm that it is of an acceptable scientific standard.

# Peer review: Reviewer response for version 1

**DOI:** 10.21956/hrbopenres.13913.r26349

**Published:** 2018-10-30

**Authors:** Helena F. Deus

**Affiliations:** 1 Elsevier, Cambridge, MA, USA

This article describes AVERT, a model - and methodology - to integrate and thus query combined clinical and environmental/weather data.

1. Use of RDF and linked data best practices because data is large and disparate is a good idea. However, there is substantial effort trying to explain why RDF/web semantics are important. This is a well established area of research, multiple conferences and journals on this topic. Benefits are mostly understood and agreed upon, no need to reiterate them here once again.
2. A weather station is both a feature (with geo coordinates) and a weather station - that’s cool; but how do you use it? It's not clear from the explanation how that benefits the query writer. Can you provide an example? 
3. FOAF is not the only ontology that they could have used - MeSH and other ontologies could have been reused (see https://bioportal.bioontology.org/) for "smoking" and many of the other terms that the authors probably ended up re-creating in their ontology
4. Where is the ontology/owl/R2ML file? Can't find the link. You can make it available through bioportal (link above) if you don't want to host it yourself. Bioportal would also find mappings to other ontologies like MeSH that are very relevant for the use case described
5. This link is broken in the article, I think there's an extra space before ".pdf" across multiple datasets.
6. The authors create an enriched csv for analysis after RDF, that’s useful. Proving a Jupyter notebook that can be used to SPARQL the data and generate the tabular format would be even better as the provenance would not be lost (which it currently is). Analysis performed on the uplifted CSV files is not FAIR as the authors claim because of this detachment of the RDF representation to the CSV uplifted version. Check out RDFlib for python access to SPARQL 
7. Very good point about the sensitivity of the data being currently protected by the presence of blockers to data accessibility. Would be interested in knowing how the authors would address this problem in the future. 
8. Evaluation is completely missing. The authors claim "facilitates quicker and more intuitive searching" but provide no timed queries to support this argument. It is "quicker" and "more intuitive" than what? Provide a comparison, a metric, something that supports this argument. There are clever ways to evaluate the intuitiveness of a SPARQL vs SQL query and the performance time but the authors didn't use any. I think this is a critical factor missing from this article. 
9. So were the authors able to test some hypothesis based on the integrated data that supports the motivating argument (KD and environmental pollutants)? What type of queries did they do? Did those give them some interesting insights? The paper is incomplete without mentioning what the integrated data was actually used for. Need more details on how this approach compares against a traditional data warehouse or any other graph database. 
10. Where's the final RDF dataset/ SPARQL endpoint? I understand that there are privacy concerns with this data but I fail to see how the data is FAIR if there's no way for other to access the data and interoperate with it. (note that FAIR does not mean open; FAIR data can be behind a paywall or a authentication service). If patient privacy is a concern, the authors can/should anonymize it. I don't think that the temporal/spacial weather data has privacy concerns. At least the SPARQL endpoint for that should be made available (or at least an RDF dump).

I confirm that I have read this submission and believe that I have an appropriate level of expertise to confirm that it is of an acceptable scientific standard, however I have significant reservations, as outlined above.

# Peer review: Reviewer response for version 1

**DOI:** 10.21956/hrbopenres.13913.r26383

**Published:** 2018-10-08

**Authors:** Nir Oren

**Affiliations:** 1 Department of Computing Science, University of Aberdeen, Aberdeen, UK

The article describes how different data sources can be transformed into RDF, over which different types of reasoning processes can be run to retrieve information. These reasoners are well developed and powerful. Once information is retrieved, it can be transformed (downlifted) for analysis by other tools. The use of RDF and open tools allows for data linkage using widely accepted standards, though caution must be taken regarding de-identification of data.

 The article provides sufficient detail for others to replicate its results, and the justification for using RDF and semantic web frameworks is well justified (namely the ability to query the *linked data* using a powerful query language). The method itself is sound, and builds on many years of research into semantic web technologies.

 The paper does not present results per se, but rather describes a method, which is easily reproducible by others given the details in the paper.

 The only concern I have is with regards to the performance of the method in the sense that running some queries over semantic web data is computationally very intensive; no discussion of the computational complexity of the approach was provided, and some indication of what queries can, or cannot be run in the context of the information that one attempts to retrieve would be useful.

I confirm that I have read this submission and believe that I have an appropriate level of expertise to confirm that it is of an acceptable scientific standard.
